# Exogenous Glutathione Alleviation of Cd Toxicity in Italian Ryegrass (*Lolium multiflorum*) by Modulation of the Cd Absorption, Subcellular Distribution, and Chemical Form

**DOI:** 10.3390/ijerph17218143

**Published:** 2020-11-04

**Authors:** Zhigang Fang, Zhaoyang Hu, Xinqiang Yin, Gang Song, Qingsheng Cai

**Affiliations:** 1 College of Life and Geographic Sciences, Kashi University, Kashi 844006, China; 2 College of Life Sciences, Nanjing Agricultural University, Nanjing 210095, China; 2016216002@njau.edu.cn (Z.H.); 2019816126@njau.edu.cn (X.Y.); songgang@jsafc.edu.cn (G.S.); qscai@njau.edu.cn (Q.C.)

**Keywords:** absorption, cadmium, chemical form, GSH, Italian ryegrass, subcellular distribution

## Abstract

Subcellular fractions and the chemical forms of cadmium (Cd) reflect its level of toxicity to plants; however, these effects of exogenous glutathione (GSH) are poorly understood. We exposed two Italian ryegrass (*Lolium multiflorum*) cultivars (IdyII and Harukaze) to 50 µM Cd or 200 µM GSH to investigate the effect of GSH on the Cd uptake, subcellular compartments, and chemical forms. Cd significantly inhibited the plant growth, while GSH supplementation decreased this inhibition. The application of GSH significantly improved the Cd concentration in the roots but reduced that in the shoots and decreased the Cd translocation from root to shoot. The Cd concentration of the root in the cell wall was increased while the concentration in the soluble fraction was decreased when supplied with GSH. The inorganic form (80% ethanol for Cd extraction) in the roots was significantly reduced when treated with GSH. The Cd form extracted by 2% acetic acid (HAC) with low toxicity and immobility were greatly increased. In leaves, the application GSH decreased in any form of Cd form extracted. In conclusion, exogenous GSH decreased the translocation of Cd and alleviated Italian ryegrass Cd toxicity by accumulating more Cd in the root cell wall and immobilizing more Cd in lower toxicity fractions.

## 1. Introduction

Cadmium (Cd), a toxic pollutant that accumulates in arable soils and water, is readily taken up by plant roots and transported to other parts, posing a hazard to the safety of the ecosystem and human health through the food chain [1,2]. Excessive Cd in plants causes severe phyto-toxicities, including leaf chlorisis, the browning of roots, and growth inhibition [3,4,5]. To prevent the negative effects of Cd on plant growth, it is necessary to alter the Cd concentration in plants. In recent years, application of exogenous materials, such as sulfide and nitrous oxide (NO), was shown to reduce Cd absorption and stimulate crop growth. These exogenous materials were characterized by the functions of modifying the subcellular distribution and chemical forms of Cd in plants, and they were expected to apply for mitigating Cd toxicity in crop plants [2,6,7].

Previous studies indicated that the subcellular distribution and chemical forms of heavy metals were associated with heavy metal tolerance and detoxification in plants [8,9,10]. The cell wall and vacuoles play important roles for Cd detoxification in different plant species. In lettuce, most Cd was shown to attach in the cell wall, and in *Potentilla griffithii* leaves, Cd was mainly isolated in vacuoles [11,12]. Additionally, Cd exhibited different chemical forms in plant organs, and this is closely related to their biotoxicity and migration capability [13]. Studies in *Phytolacca americana* and *Porphyra yezoensis* showed that the inorganic form of Cd (extracted by 80% ethanol) was more toxic to plants, while the Cd compartmentalized in phosphate Cd (extracted by 2% acetic acid, HAC) and undissolved Cd in protein (extracted by 1 M NaCl) were less toxic [14,15].

Reduced glutathione (γ-Glu-Cys-Gly, GSH), a major low molecular weight thiol compound in plant cells, is involved in the cellular defense against the toxic action of salinity and metal cations [16,17]. GSH has a crucial role to detoxify heavy metals. As the precursor of phytochelatins (PCs), GSH is also involved in the sequestration of heavy metals in vacuole [18,19]. Researchers reported that exogenous GSH alleviated Cd toxicity in plants by reducing or maintaining Cd uptake [20,21,22], while few studies have examined the subcellular distributions and chemical forms of Cd with GSH treatment [23].

Italian ryegrass (*Lolium multiflorum* Lam.), an annual grass species with wide geographic distribution and high biomass, can be easily cultivated in southern China [24]. It was introduced to southern China during the winter for relieving green fodder shortages in the early 1990s, and is in development for phytoremediation and bio-ethanol production due to the accumulating ability of heavy metals (including Cu, Zn, and Cd) in the roots and shoots and the high-potential conversion efficiency of bio-ethanol [25,26,27]. Our previous studies reported that high Cd stress caused serious toxicity in Italian ryegrass and resulted in growth inhibition [28]. However, little information to demonstrate the potential mechanisms of Cd-detoxification by the addition of GSH is available on Cd detoxification associated with the subcellular distribution of the chemical forms of Cd with the exogenous GSH treatments [29]. In this work, we aimed to investigate the Cd uptake, subcellular distribution, and chemical forms in Italian ryegrass in response to Cd stress applied with exogenous GSH. The results will deepen the understanding of the strategy through which exogenous GSH mitigates plant Cd stress and contributes to remediating the Cd from agricultural environments.

## 2. Materials and Methods

### 2.1. Plant Materials and Growth Conditions 

Two Italian ryegrass cultivars differing in Cd tolerance, namely IdyII (relative Cd tolerance) and Harukaze (relative Cd sensitive), were selected according to our previous study [28]. Seeds were sterilized with 10% H_2_O_2_ for 15 min, rinsed thoroughly with distilled water and germinated at 25 °C in the dark. The 6-day-old seedlings with uniform size were transferred to a 1 L plastic beaker filled with 1/4 Hoagland solution (pH 6.5). The seedlings were placed in a growth chamber with the following conditions: 12 h light/dark cycle with photosynthetic photon flux density 300 μmol m^−2^ s^−1^, temperature 25/20 °C (day/night) and relative humidity 65 ± 5%.

### 2.2. Experiment Design

After 10 days of the acclimatization phase, Cd (CdCl_2_·2.5H_2_O, analytical grade) at a concentration of 50 μM with or without 200 μM GSH was added to the corresponding containers to form four groups: (1) CK (did not add anything to 1/4 Hoagland solution), (2) Cd (added 50 μM Cd to 1/4 Hoagland solution), (3) Cd + GSH (added 50 μM Cd and 200 µM GSH to 1/4 Hoagland solution), and (4) GSH (added 200 µM GSH to 1/4 Hoagland solution). The GSH and Cd concentration was selected based on our preliminary studies [28]. The nutrient solution was renewed every 3 days during the experiment. Each group was conducted in six replicates. Seedlings were collected after 12 days of treatment and were then divided into two groups (three replicates as one group). One group was determined for plant biomass and Cd concentration, while the other was determined for Cd concentration in different subcellular fractions and chemical forms.

### 2.3. Sampling and Subcellular Fractions Fractionation

The plant samples were soaked in 20 mM ethylenediaminetetraacetic acid disodium salt (Na_2_-EDTA) for 15 min to remove metals on the root surfaces, rinsed with distilled water, and then were separated into roots and shoots. Subsequently, the samples were oven-dried at 70 °C to a constant weight. The dried tissues were weighed, and used to determine the Cd concentration.

Our investigation of the subcellular distribution of Cd in plant tissues was performed as described by Weigel and JaGer with minor modifications [30]. The uniformly treated seedlings were gently uprooted from solution, washed with distilled water, separated into roots and shoots, weighed (0.3 g), and homogenized in ice-cold medium containing 0.25 M sucrose, 50 mM Tris–HCl buffer solution (pH 7.5), and 1.0 mM DL-dithioerythritol. The cells were separated using the gradient centrifugation technique at 4 °C into three fractions: cell wall, soluble fraction, and organelles. The homogenate was centrifuged at 765 g for 20 min, and the precipitation was designated as the ‘cell wall fraction’, mainly consisting of cell walls and cell wall debris. The resulting supernatant solution was further centrifuged at 19,118× *g* for 30 min. The resultant deposition and the supernatant solution were referred to as the ‘organelle fraction’ and ‘soluble fraction’, respectively. The cell wall and cell organelle fractions were dried at 70 °C to a constant weight, and left to digest, to determine the Cd concentration with soluble fraction.

### 2.4. Extraction of Cd in Different Chemical Forms

Chemical forms of Cd in the roots and shoots were sequentially extracted according to the method of Mwamba and others as follows [31]. We used (1) 80% ethanol, extracting the inorganic Cd and giving priority to nitrite, chloride, and aminophenol thorium (F ethanol); (2) deionized water, extracting the water soluble Cd-organic acid salts (F d-H_2_O); (3) 1 M of NaCl, extracting the Cd integrated with pectate and protein integrated Cd (F NaCl); (4) 2% Acetic acid (F HAC), extracting the sparingly soluble or insoluble phosphate bound Cd (F HAC); (5) 0.6 M HCl, extracting Cd oxalates (F HCl); and (6) the Cd in the residue (F Residue). 

### 2.5. Cd Concentration, Translocation Factor (TF), and Tolerance Index (TI)

The samples (roots, shoots, cell wall, cell organelle fractions, and Cd in the residue) were digested with 5 mL of HNO_3_ + HClO_4_ (85:15, v/v) using a DigiBlock ED54-iTouch Digester (LabTech, Beijing, China). The Cd concentration was determined using an inductively coupled plasma optical emission spectrometer (ICP-OES, Optima 2100DV, Perkin Elmer, Gaithersburg, MD, USA) at the wavelength of 226.502.

The translocation factor (TF) and the tolerance index (TI) were determined according to the method of Chen et al. (2011) [32]. TF = Cd_shoot_/Cd_root_, where Cd_shoot_ and Cd_root_ indicate the Cd concentration in the shoots and roots, respectively. TI = biomass_treatment_/biomass_control_.

### 2.6. Statistical Analysis

The data are the mean values ± SE (standard errors) of three replicates. Statistical analyses were performed with analysis of variance (ANOVA) using SPSS 20.0 software. To test the main characteristics that affected the TI of the roots, the proportion of Cd in the root cell wall and chemical form of Cd in the plant roots were determined via Pearson’s correlation analysis.

## 3. Results

### 3.1. Effect of GSH on the Growth of Italian Ryegrass Seedlings under Cd Stress

Cd exposure significantly inhibited the biomass of the roots and shoots (Table 1), being more pronounced in Harukaze. The addition of GSH significantly alleviated Cd-induced growth inhibition, and the increase in root biomass reached 54% in IdyII and 138% in Harukaze (Table 1). The Tls (root and shoot) of IdyII were higher than those of Harukaze under Cd stress, and a similar trend was exhibited with the supplementation of GSH (Table 1). No significant growth change was shown when exogenous GSH was added into the control.

### 3.2. Effect of GSH on Cd Uptake and Translocation in Italian Ryegrass Seedlings

**Figure 1:**
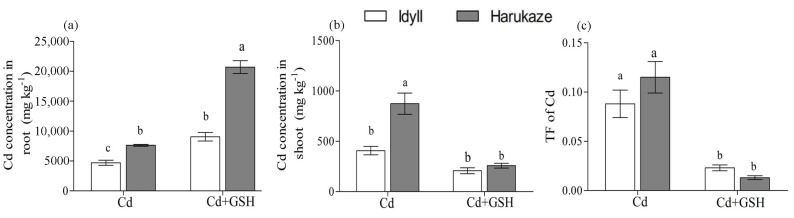
The effect of GSH on the Cd concentration in plant roots (**a**), shoots (**b**), and translocation factor (TF) (**c**) exposed to Cd stress. Values are means ± SE (*n* = 3). Different letters in the column indicate significant differences among the treatments at *p* < 0.05. Cd, 50 µM Cd; Cd + GSH, and 50 µM Cd with 200 µM GSH. Cd in CK and GSH was below the detection.

The results of applied exogenous GSH on the Cd absorption in plant tissue are shown in Figure 1. The Cd concentration of the roots and shoots and were analyzed under Cd stress and Cd together GSH. Surprisingly, exogenous GSH significantly enhanced the Cd accumulation in the roots of Cd-treated ryegrass cultivars, producing a 92% increase in IdyII and 171% in Harukaze (Figure 1a). This increase was not found in the shoots of IdyII and Harukaze (Figure 1b). Contrarily, a reduced Cd concentration in Cd-treated ryegrass shoots were shown when exogenous GSH was applied (Figure 1b). These opposite effects in the roots and shoots demonstrated that exogenous GSH restricted Cd translocation from the roots to shoots (Figure 1c).

### 3.3. Effect of GSH on Subcellular Distribution of Cd in Italian Ryegrass Roots and Shoots

**Figure 2:**
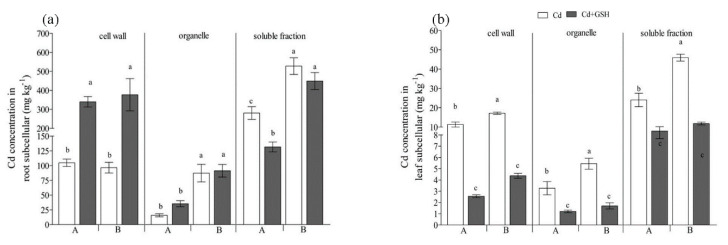
The effect of GSH on the subcellular distribution of Cd in Italian ryegrass roots (**a**) and leaves (**b**) exposed to Cd stress. The capital letters A and B on the horizontal axis represent IdyII and Harukaze, respectively. Cd, 50 µM Cd; Cd + GSH, 50 µM Cd with 200 µM GSH; Values are means ± SE (*n* = 3). Different letters in the same subcellular fraction indicate significant differences among the treatments at *p* < 0.05.

The majority of Cd accumulated in the soluble fraction when the seedlings treated with Cd (Figure 2a), representing 69.7% and 74.3% of the total Cd in root cells for IdyII and Harukaze, respectively (Table 2). By contrast, a smaller amount of Cd was observed in the organelle fraction (4.1% and 12.1% for IdyII and Harukaze, respectively) and cell wall (26.3% and 13.6% for IdyII and Harukaze, respectively) (Table 2). The Cd in the soluble and organelle fraction exhibited significant differences between the two cultivars (Figure 2a). The application of GSH decreased the Cd amounts in the soluble fraction, being more distinct in IdyII. Contrarily, Cd in the root cell wall was greatly increased in the two cultivars with supplemented GSH (Figure 2a), representing 67.0% and 40.2% of the total Cd for IdyII and Harukaze, respectively (Table 2). Consequently, significant differences in the distribution of Cd in the root cell wall and soluble fraction were observed between the two cultivars in the presence of GSH (Table 2).

In the leaf cells, Cd predominated in the soluble fraction (Figure 2b), ranging from 62.0% to 70.0% of the total Cd in the leaves for IdyII and Harukaze (Table 2). The Cd concentrations in the fractions (cell wall, organelle, and soluble) of the Harukaze leaf cells were significantly higher than the corresponding of IdyII under Cd stress (Figure 2b). The application of GSH reduced the Cd concentration in any fraction (Figure 2b). Consequently, compared with Cd stress, the distribution of Cd in the leaf soluble fraction of IdyII was significantly increased. By contrast, a significant reduction in the distribution of Cd in the cell wall was observed in IdyII. No change in the distribution of Cd in leaf cells was demonstrated in Harukaze between the Cd and GSH treatments (Table 2).

### 3.4. Effect of GSH on Chemical Forms of Cd in Italian Ryegrass Roots and Shoots

**Figure 3:**
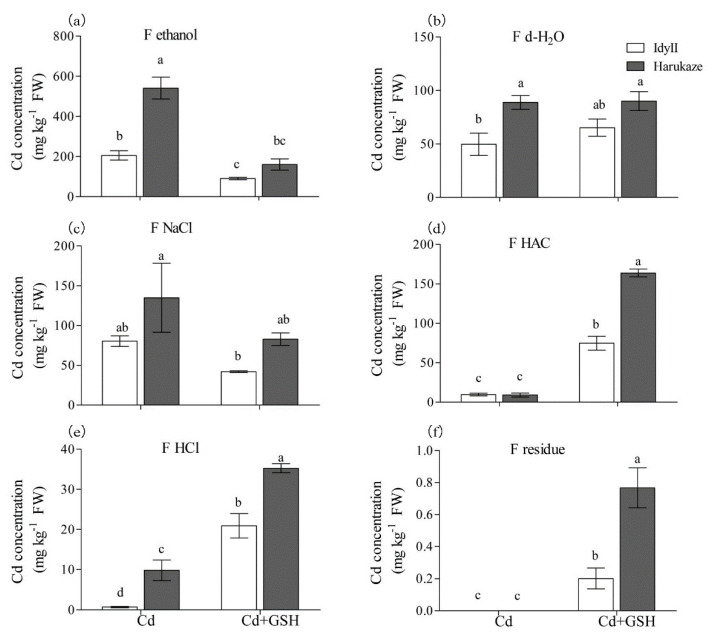
The effect of GSH on the Cd concentration of chemical forms in Italian ryegrass roots exposed to Cd stress. Values are means ± SE (*n* = 3). Cd, 50 µM Cd; Cd + GSH, 50 µM Cd with 200 µM GSH; Cd in different chemical forms was extracted successively by the following extraction solutions: (**a**) 80% ethanol (F ethanol); (**b**) d-H_2_O (F H2O); (**c**) 1 M NaCl (F NaCl); (**d**) 2% acetic acid (HAC) (F NaCl); (**e**) 0.6 M HCl (F HCl); (**f**) residue (F R). Different letters in the same chemical forms indicate significant differences among the treatments at *p* < 0.05.

**Figure 4:**
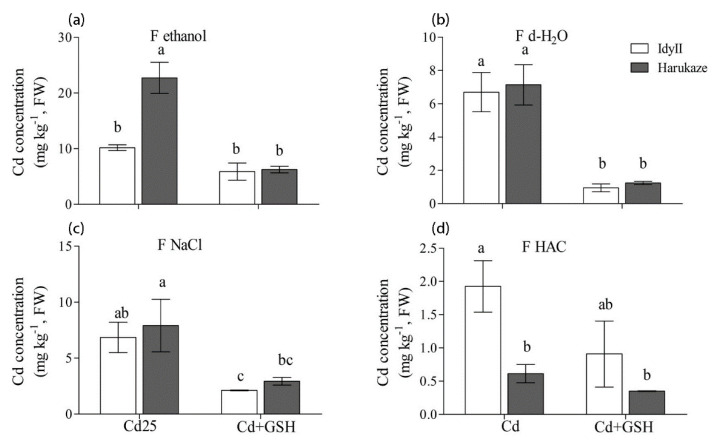
The effect of GSH on the Cd concentration of chemical forms in Italian ryegrass leaves exposed to Cd stress. The values are means ± SE (*n* = 3). Cd, 50 µM Cd; Cd + GSH, 50 µM Cd with 200 µM GSH; Cd in different chemical forms was extracted successively by the following extraction solutions: (**a**) 80% ethanol (F ethanol); (**b**) d-H_2_O (F H_2_O); (**c**) 1 M NaCl (F NaCl); (**d**) 2% HAC (F NaCl). Different letters in the same chemical forms indicate significant differences among the treatments at *p* < 0.05.

The Cd concentrations of different chemical forms in ryegrass roots and shoots are shown in Figure 3 and Figure 4. Overall, Cd exposure resulted in a predominant form (using 80% ethanol for Cd extraction), which accounted for 59.3% of the total amount in IdyII and 69.7% for Harukaze (Figure 3a, Table 3). By contrast, a minor part the concentration of Cd extracted by HAC (1.2% for IdyII and 2.8% for Harukaze) and 0.6 M HCl (0.2% for IdyII and 1.4% for Harukaze) were noted (Figure 3a,d,e, Table 3). We found that the amounts of Cd extracted by 80% ethanol, d-H_2_O, and 0.6 M HCl in Harukaze were significantly higher than the corresponding values for IdyII under Cd stress (Figure 3a–c). However, only the proportion of 0.6 M HCl-extracted Cd exhibited significant difference between the two cultivars (Table 3). The application of GSH significantly reduced the concentration of Cd extracted by 80% ethanol, and was more distinct in Harukaze. On the other hand, the amounts of Cd extracted by 2% HAC, 0.6 M HCl, and the residue were greatly increased between the two cultivars, and the amounts of Cd in Harukaze were significantly higher than those of IdyII (Figure 3a,d–f). Thus, compared with Cd stress, the proportion of Cd extracted with 80% ethanol was reduced by 28% in IdyII and 40% in Harukaze with supplemented GSH, while the proportion of Cd extracted with HAC increased by 22% in IdyII and 30% in Harukaze (Table 3). A significant difference was exhibited in the proportion of the Cd form extracted by HAC between the two cultivars in the presence of GSH (Table 3). 

In leaves, the concentration of Cd extracted by 80% ethanol in Harukaze was significantly higher than these of IdyII under Cd stress, while a reverse trend was shown in the amounts of Cd extracted by HAC (Figure 4a,d). Application of GSH resulted in a great reduction in the concentration of Cd extracted by d-H_2_O and 1 M NaCl in two cultivars, and similar results were seen in the amounts of Cd extracted by 80% ethanol of Harukaze (Figure 4a–c). The proportion of Cd extracted by 80% ethanol, d-H_2_O and 1 M NaCl showed no difference between the two cultivars, respectively (Table 3). The proportion of Cd extracted by HAC in IdyII was significantly higher than that of Harukaze.

### 3.5. Correlations Analysis among TI, the Proportion of Cd in the Root Cell Wall and the Proportion of the Cd Form in the Roots

Table 4 showed that TI was significantly positively correlated with the proportion of Cd in the root cell wall, the proportion of Cd extracted by HAC (F HAC%), 0.6 M HCl (F HCl%), and the residue (F R%). The proportion of Cd extracted by 80% ethanol (F ethanol%) showed a significantly negative correlation with F HAC%, F HCl%, and F R%. Similarly, significantly positive correlations were also exhibited among RCW% and the proportion of Cd extracted by d-H_2_O (FH_2_O%), 2% HAC, and 0.6 M HCl. 

## 4. Discussion

The plant biomass and TI were used to evaluate the Cd toxicity and tolerance in different species and cultivars [33,34]. In the current study and previous study, higher plant growth inhibition was exhibited in Harukaze compared with in IdyII (Table 1), implying that IdyII is more tolerant to Cd than Harukaze [35]. The addition of GSH in Cd-exposure seedlings caused the marked increment of plant biomass and reduction in Cd concentration in plant shoots, particularly for Harukaze (Table 1, Figure 1). Similar results were observed in rice treated with 5 and 50 μM GSH [36], suggesting that exogenous GSH enhanced plant tolerance to Cd and alleviated Cd toxicity partly through reducing Cd uptake in plant shoots. 

In this study, we also found that the exogenous application of GSH enhanced ryegrass tolerance to Cd. This enhanced Cd tolerance was not associated with the decrease in Cd uptake in roots. Conversely, an increase in Cd concentration was detected in the roots (Figure 1). Similarly, exogenous GSH that promoted Cd accumulation in root and alleviated its toxicity was reported in *Populus × canescens* and *Brassica campestris* L. seedlings [22,37]. In barley, supplementation of 20 mg L^−1^ GSH significantly reduced the Cd accumulation in leaves and roots [20]. Exogenous GSH application did not result in an observed change in the Cd concentration in Cole (*Brassica campestris* L. *Cruciferae*) roots and leaves exposed to Cd stress [38]. Other studies showed that the increase in root exudates (polysaccharides, proteins, and organic acids) and the reducing pH in culture solution accounted for the increase in Cd accumulation in rice roots [39,40]. The effects of GSH on Cd accumulation in plants appears to be complicated and dependent on the plant species and tissues.

Plants exhibit a variety of strategies to cope with excess Cd, including vacuolar sequestration, immobilization by cell walls, and complexation with thiol compounds. Low uptake and low translocation from the root to shoot of Cd were regarded as the major strategies for plants regarding Cd tolerance [41]. The opposite effects of Cd concentration in ryegrass roots and shoots demonstrated that exogenous GSH decreased the Cd translocation from the roots to shoots, which was more pronounced in Harukaze (Figure 1), indicating that Harukaze, with higher tolerance for applied GSH, was more beneficial to lower the TF (Figure 1, Table 1). On the other hand, subcellular partitioning of Cd in plants was also the approach to enhance Cd tolerance and detoxification in response to Cd stress [42]. 

In the present study, with Cd treatment, the Cd concentrations in thee subcellular fractions followed the order of soluble fraction > cell wall > organelle (Figure 2). The results are consistent with the previous report on the soybean cultivars under Cd stress [43]. By contrast, the Cd amounts and the proportion of Cd in ryegrass root cell walls were significantly increased by the application of 200 µM GSH (Figure 2a, Table 2), implying that Cd^2+^ was mainly deposited in the root cell wall and that the transmembrane transport was limited, which was partly confirmed by the Cd amounts in the shoots and TFs (Figure 1b,c). 

A recent study showed that applying exogenous GSH in hydroponic solution was found to activate and retain Cd outside in *Brassica napus* and *Arabidopsi* roots, particularly in cell wall fractions, rather than the binding of GSH [44,45]. They also demonstrated that the application of exogenous GSH in solution was more effective at increasing the Cd accumulation in *Arabidopsis thaliana* roots compared with the enhancement of endogenous GSH [44]. Secondly, the synthesized PCs resulted in the mitigation of Cd activity through complexation and compartmentalization in tissues, and, consequently, the Cd toxicity in the plants was reduced [46]. 

Based on this evidence, we propose that exogenous GSH activated the ability to retain Cd in ryegrass roots and that the root cell wall played a vital role to enhance the Cd tolerance through the application of GSH. This deduction is partly supported by the significant positive correlation between TI and the proportion of root cell wall distribution of Cd (Table 4). Previous studies revealed that the root cell wall contained polysaccharides and protein, providing negative charge sites on their surface binding Cd ions and restricting their transportation across the cytomembrane, which is often induced by environmental factors [47]. Research proved that pectin methylesterase (PME) is involved in Cd retention in the root cell wall by regulating the metabolites of polysaccharides [48]. In future studies, it is necessary to clarify the effects of exogenous GSH on the metabolites of polysaccharides in ryegrass roots. In view of the results of this study, the difference in the proportion of Cd retention in the root cell wall between IdyII and Harukaze was the cause of differences in cadmium tolerance with the application of GSH.

Cadmium storage in the soluble fraction (mainly in vacuoles) was regarded as an essential detoxification response to Cd for plant leaves [49]. We found the majority of Cd was present in the soluble fraction in Italian ryegrass leaves (Figure 2b), which consisted mostly of vacuoles and acted as the predominant site of Cd binding in leaf cells [31]. GSH treatments significantly reduced the Cd concentration in any subcellular fraction of leaves, and the proportion of the soluble fraction of IdyII was significantly increased by the addition of GSH (Table 2), implying that more Cd ions could be compartmentalized in the vacuole of IdyII leaves. Our results suggest that vacuolar compartmentalization is a predominant strategy for Italian ryegrass leaves in detoxification with the addition of GSH. 

The chemical forms of Cd are closely related to their biological toxicity. For instance, Cd bonds in the inorganic form (extracted by 80% ethanol) and water-soluble form (extracted by deionized water) have greater harmful effects to cells than other chemical forms due to the higher capacity to migrate [31]. Cd extracted by 80% ethanol was predominant in *Bechmeria nivea* L. Gaud roots under Cd stress, which mainly consisted of inorganic forms bound to the compounds with high mobility and toxicity [50]. Similar results were also observed in Italian ryegrass root under Cd stress, the toxicity easily appeared in Harukaze with lower TI (Table 1), which was partly supported by the significant negative correlation between the root TI and the proportion of Cd extracted by 80% ethanol (Table 4). The proportion of Cd bonded to undissolved phosphate (Cd extracted by 2% HAC) with low mobility and toxicity showed an increase in the *Exophiala pisciphila mycelia* root under Cd stress [51]. 

In the present study, compared with Cd stress, the amounts and the proportions of the undissolved phosphate (extracted by 2% HAC) and low-bioavailable compounds (extracted by 0.6 M HCI) were markedly increased in Italian ryegrass root by the application of GSH (Figure 4d–f; Table 3). These results indicate that undissolved phosphate and oxalate acids should be important as metal ligands in Cd accumulation and detoxification. Those two metal ligands are proposed to enhance the Cd tolerance in Italian ryegrass when GSH was applied, which is supported by the significant positive correlation between the root TI and the proportion of Cd extracted by HAC and Cd extracted by 0.6 M HCl in plant roots (Table 4). 

In the leaves of both cultivars, Cd in inorganic forms was dominant for any treatment. Cd in pectinates and protein-integrated Cd (extracted by 1 M NaCl) was dominant in the leaves of *Phytolacca americana* L under Cd stress [14]. Another study reported that Cd bonded to undissolved phosphate was enhanced in the leaves of *Porphyra yezoensis* with increasing Cd levels [15]. These results suggest that the different ligands involved in the Cd toxicity in plant leaves depended on the plant species, Cd strength, and culture conditions. In this study, the proportion of Cd bonded to undissolved phosphate in IdyII leaves was significantly higher than that of Harukaze in the presence of GSH, indicating that this strategy could be taken against Cd toxicity in IdyII.

## 5. Conclusions

In conclusion, GSH treatment effectively alleviated the Italian ryegrass growth inhibition by Cd. At the subcellular level, the application of GSH resulted in the strong ability to retain Cd in the root cell wall of IdyII. On the other hand, GSH treatment resulted in the largest proportion of Cd bonded to undissolved phosphate (extracted by 2% HAC) with immobilization and less toxicity in the two cultivar roots with more effects in Harukaze. The applied exogenous GSH alleviated the Cd toxicity in Italian ryegrass though modulation in the subcellular distribution and chemical form of Cd, which were likely implemented for limiting Cd translocation from the root to shoot. The results of the present study provide the basic information for alleviating Cd toxicity and can contribute to the cultivation of forage crops used as bioenergy crops in heavily Cd-polluted environments with the application of GSH.

## Figures and Tables

**Table 1:** The effect of glutathione (GSH) on the plant biomass of Italian ryegrass seedlings under cadmium (Cd) stress.

Cultivar	Treatment	Root DW/mg	Root Tl	Shoot DW/mg	Shoot TI
IdyII	CK	22.51 ± 2.77 a	1.00	123.98 ± 10.4 a	1.00
	Cd	13.30 ± 1.57 c	0.59	80.48 ± 8.41 c	0.65
	Cd + GSH	20.54 ± 1.71 ab	0.91	104.42 ± 5.65 ab	0.84
	GSH	24.79 ± 1.87 a	1.10	122.38 ± 5.55 a	0.99
Harukaze	CK	15.29 ± 0.99 bc	1.00	84.00 ± 5.05 bc	1.00
	Cd	5.46 ± 0.58 d	0.36	35.20 ± 3.62 e	0.42
	Cd + GSH	13.02 ± 2.43 c	0.85	59.41 ± 6.82 d	0.71
	GSH	14.72 ± 1.47 c	0.96	94.83 ± 3.90 bc	1.12

DW, dry weight; CK, control; Cd, 50 µM Cd; Cd + GSH, 50 µM Cd with 200 µM GSH; TI indicates tolerance index; Values are means ± standard error (SE) (*n* = 3). Different letters in the same column indicate significant differences among the treatments at *p* < 0.05.

**Table 2:** The effect of GSH on the Cd subcellular distribution in the roots and leaves of two Italian ryegrass cultivars exposed to Cd.

Cultivar	Treatment	Root			Leaf		
		Cell Wall%	Organelle%	Soluble Fraction%	Cell Wall%	Organelle%	Soluble Fraction%
IdyII	Cd	26.28 ± 1.01 c	4.06 ± 0.93 c	69.66 ± 1.87 a	29.50 ± 1.53 a	8.53 ± 1.31 a	61.97 ± 2.81 b
	Cd + GSH	66.98 ± 2.50 a	6.94 ± 0.79 bc	26.08 ± 1.95 c	20.58 ± 2.53 b	9.67 ± 1.28 a	69.75 ± 3.22 a
Harukaze	Cd	13.56 ± 0.22 d	12.11 ± 1.38 a	74.33 ± 1.58 a	25.14 ± 0.21 ab	7.92 ± 0.47 a	66.95 ± 0.26 ab
	Cd + GSH	40.21 ± 5.49 b	10.27 ± 2.04 ab	49.52 ± 5.06 b	24.46 ± 0.87 ab	9.38 ± 0.87 a	66.16 ± 0.67 ab

Cd, 50 µM Cd; Cd + GSH, 50 µM Cd with 200 µM GSH; Different letters in the same column indicate significant differences among the treatments at *p* < 0.05.

**Table 3:** The effect of GSH on the distribution of each chemical form of Cd in Italian ryegrass exposed to Cd stress.

Tissue	Cultivar	Treatment	F Ethanol%	F d-H_2_O%	F NaCl%	F HAC%	F HCl%	F Residue%
Root	IdyII	Cd50	59.25 ± 5.42 a	14.45 ± 3.03 b	23.31 ± 1.91 a	2.79 ± 0.49 c	0.20 ± 0.04 c	0 c
		Cd50 + GSH	30.96 ± 1.00 b	22.07 ± 0.95 a	14.51 ± 1.12 b	25.36 ± 1.01 b	7.05 ± 0.45 a	0.2 ± 0.06 b
	Harukaze	Cd50	69.72 ± 2.52 a	11.59 ± 1.1 b	16.18 ± 3.92 ab	1.15 ± 0.26 c	1.36 ± 0.47 b	0 c
		Cd50 + GSH	29.69 ± 3.56 b	17.11 ± 2.25 ab	15.49 ± 0.44 b	30.92 ± 1.5 a	6.65 ± 0.27 a	0.77 ± 0.13 a
Leaf	IdyII	Cd50	40.84 ± 6.29 a	25.69 ± 2.32 a	26.13 ± 3.14 a	7.34 ± 0.88 a	-	-
		Cd50 + GSH	58.25 ± 9.64 a	9.86 ± 2.67 c	22.36 ± 3.14 a	9.67 ± 2.03 a	-	-
	Harukaze	Cd50	59.46 ± 7.17 a	18.36 ± 2.06 b	20.51 ± 5.97 a	1.67 ± 0.49 b	-	-
		Cd50 + GSH	57.93 ± 0.58 a	11.71 ± 1.22 bc	27.05 ± 1.07 a	3.31 ± 0.33 b	-	-

Cd, 50 µM Cd; Cd + GSH, 50 µM Cd with 200 µM GSH; different letters in the same column indicate significant differences among the treatments at *p* < 0.05.

**Table 4:** The correlation coefficients among the root TI, the proportion of Cd in root cell wall and chemical form.

Index	Tl	RCW%	F Ethanol%	F H_2_O%	F NaCl%	F HAC%	F HCl%	F Residue%
Tl	1							
RCW%	0.860 **	1						
F ethanol%	–0.774 **	–0.812 **	1					
F H_2_O%	0.545	0.776 **	–0.805 **	1				
F NaCl%	–0.205	–0.316	0.254	–0.255	1			
F HAC%	0.767 **	0.753 **	–0.954 **	0.663 *	–0.460	1		
F HCl%	0.698 *	0.791 **	–0.891 **	0.700 *	–0.637 *	0.954 **	1	
F Residue%	0.747 **	0.550	–0.823 **	0.439	–0.404	0.913 **	0.822 **	1

TI, RCW, F ethanol%, F H_2_O%, F NaCl%, F HAC%, F HCl%, and F Residue% indicate the tolerance index, the proportion of Cd in root cell wall, the proportion of F ethanol, the proportion of F H_2_O, the proportion of F NaCl, the proportion of F HAC, the proportion of F HCl, and the proportion of F Residue, respectively. * *p* < 0.05, ** *p* < 0.01.
